# Exercise maintains bone homeostasis by promoting osteogenesis through STAT3

**DOI:** 10.7150/ijbs.82744

**Published:** 2023-04-02

**Authors:** Xiangru Huang, Yanfei Zhu, Siyuan Sun, Xin Gao, Yiling Yang, Hongyuan Xu, Anting Jin, Yuanqi Liu, Hanbing Jia, Qinggang Dai, Lingyong Jiang

**Affiliations:** 1 Center of Craniofacial Orthodontics, Department of Oral and Cranio-maxillofacial Surgery, Shanghai Ninth People's Hospital, Shanghai Jiao Tong University School of Medicine; College of Stomatology, Shanghai Jiao Tong University; National Center for Stomatology; National Clinical Research Center for Oral Diseases; Shanghai Key Laboratory of Stomatology; Shanghai Research Institute of Stomatology.; 2 The 2nd Dental Center, Ninth People's Hospital, Shanghai Ninth People's Hospital, Shanghai Jiao Tong University School of Medicine; College of Stomatology, Shanghai Jiao Tong University; National Center for Stomatology; National Clinical Research Center for Oral Diseases; Shanghai Key Laboratory of Stomatology; Shanghai Research Institute of Stomatology.

**Keywords:** exercise, mechanical force, STAT3, running wheel, tail suspension, bone homeostasis

## Abstract

Bone exhibits changes in density, strength, and microarchitecture in relation to mechanical loading mediated by exercise. Appropriate exercise maintains bone homeostasis, while the absence of exercise leads to disuse bone loss. However, the acting mechanism of mechanotransduction in bone remains unclear. We performed the running-wheel exercise and tail suspension model to study the effects of exercise on bone metabolism, and found that osteoblastic Signal transducer and activator of transcription 3 (STAT3) activity was closely related to exercise-induced bone mass and metabolism changes. With the Flexcell tension-loading system* in vitro*, mechanical force promoted STAT3 activity, which was accompanied by increased osteoblastic differentiation of the bone marrow mesenchymal stem cells (BMSCs). In contrast, the inhibition of STAT3 phosphorylation blocked force-induced osteoblastic differentiation. Furthermore, pharmacological inactivation of STAT3 impaired the increase in exercise-induced bone mass and osteogenesis. With an inducible conditional deletion mouse model, we found that the osteoblast lineage-specific Stat3 deletion could also block force-induced osteoblastic differentiation *in vitro* and impair exercise-promoted bone mass and osteogenesis *in vivo*. This confirmed the crucial role of osteoblastic STAT3 in exercise-mediated bone metabolism. Finally, colivelin, a STAT3 agonist, promoted osteoblastic differentiation *in vitro* and partly rescued exercise loss-induced disuse bone loss by improving osteogenesis in the tail suspension model. Taken together, our study revealed the essential role of STAT3 in maintaining exercise-mediated bone homeostasis. In addition, STAT3 might act as a potential target for osteoporosis caused by exercise loss.

## Introduction

Exercise-mediated mechanical force plays an essential role in maintaining bone homeostasis. According to Julius Wolff's law, sufficient mechanical force induces potent anabolism that strengthens bone architecture and increases bone mass 1, 2. Relevantly, a lack of exercise results in bone loss and a reduction in bone strength, which inevitably happens to paralytic patients and astronauts in weightlessness 3, 4. Hence, clarifying the underlying mechanisms of exercise-mediated bone homeostasis would be a key factor in treating osteoporosis caused by loss of exercise.

Bone homeostasis is the balance between bone formation and bone resorption 5, 6. An imbalance in bone formation and bone resorption leads to dysfunction and incomplete bone. It has been reported that mechanical loading promotes bone formation 7, 8. Osteoblasts, as bone-forming cells, are derived from bone marrow mesenchymal stem cells (BMSCs) 9. BMSCs not only are the major stem cell for osteogenesis but can also transfer the mechanical signals into biological signals and coordinate bone resorption and formation 7, 10. However, the underlying molecular mechanisms by which BMSCs regulate exercise-driven bone formation remain less defined.

Several mechanosensory molecules participate in bone mechanotransduction, including YAP, Piezo1, and follistatin-like 3 11-13. Signal transducer and activator of transcription 3 (STAT3) might emerge as a significant mediator of various aspects of mechanotransduction. Previous studies demonstrated that transcriptional activation factor 3 (STAT3) is critical for skeletal growth and bone homeostasis and might be a potential mechanical-sensitive molecular target for regulating bone homeostasis 14-18. Thus, this research intended to concentrate on how STAT3 regulates bone homeostasis under mechanical force mediated by exercise.

In this study, a running-wheel model and a tail-suspension model were used to study bone metabolism and the role of STAT3 with and without exercise. We revealed that STAT3 activity was closely related to exercise-induced bone mass and metabolism changes. Meanwhile, mechanical force *in vitro* promoted osteoblastic differentiation of BMSCs* via* STAT3. Both pharmacological inhibition and osteoblast lineage-specific deletion of STAT3 impaired exercise-induced osteogenesis and bone mass increase. More importantly, we found that STAT3 might serve as a potential target to treat bone loss due to exercise loss by promoting osteogenesis.

## Results

### Exercise promoted osteogenesis and STAT3 phosphorylation

**Figure 1:**
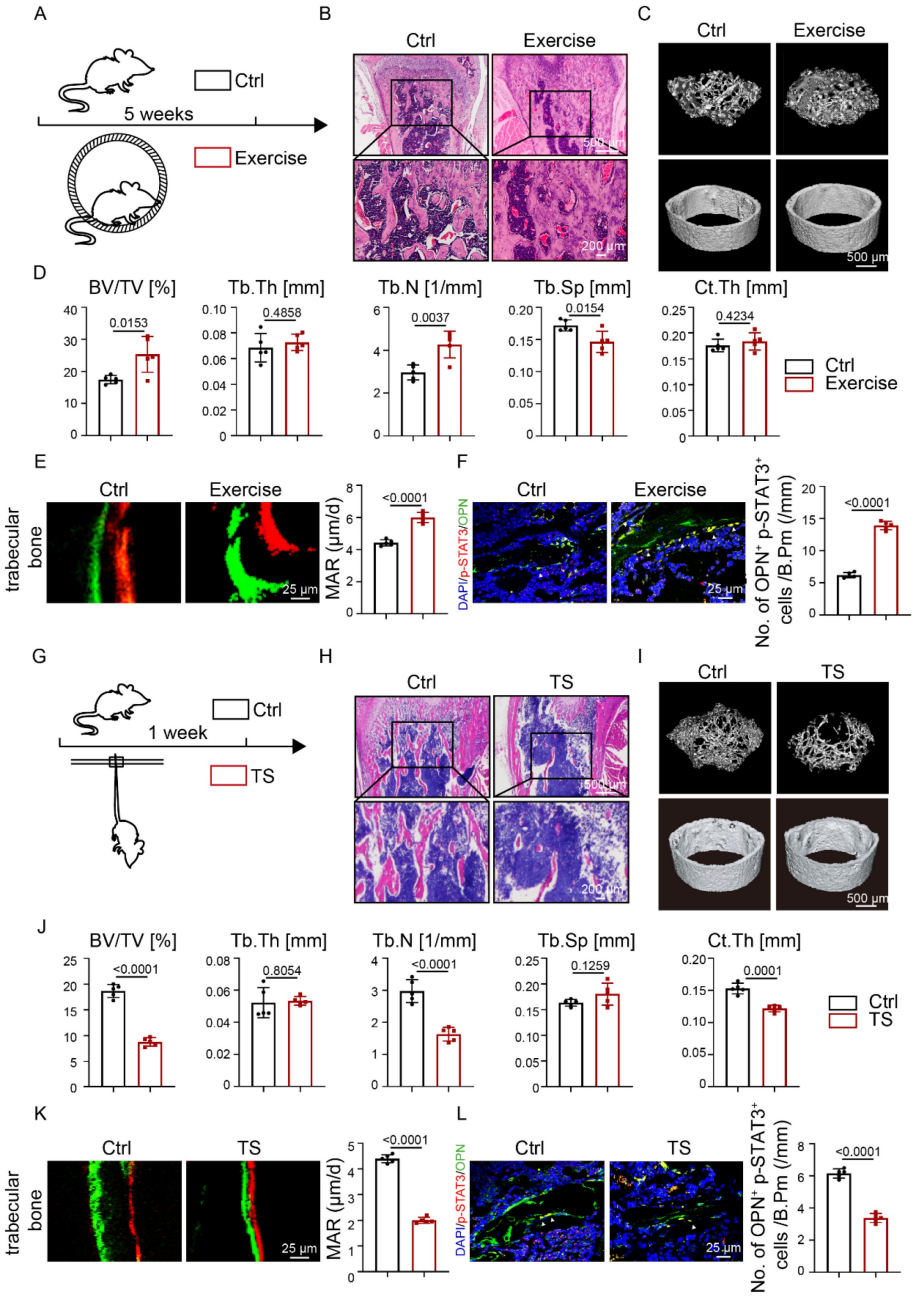
** Osteoblastic STAT3 was closely related to exercise-mediated mechanical force* in vivo.* A.** Experimental outline of the running-wheel exercise test. Skeletal parameters were measured in C57BL/6 mice that had access to exercise wheels for 5 weeks or not. **B.** H&E staining of the femurs of the control and exercise mice. **C.** Three-dimensional micro-CT reconstruction images of femurs from the control and exercise mice. The *top panel* shows trabecular bone, and the *bottom panel* represents cortical bone. Representative examples are shown. **D.** Quantitative microarchitectural parameters of micro-CT: BV/TV, Tb.Th., Tb.N., Tb.Sp. and Ct.Th. Five pairs of control and exercise mice were included in the measurement. **E.** Representative images of dual calcein-alizarin red S labeling of trabecular bone from the control and exercise mice acquired 5 weeks after *in vivo* exercise treatment and quantification of mineral apposition rate. **F.** Representative images of the immunofluorescence for p-STAT3 in trabecular bone from the control and exercise mice. **G.** Experimental outline of the tail suspension test. Skeletal parameters were measured in C57BL/6 mice that had tail-suspension treatment for 1 week or not. **H.** H&E staining of the femurs of the control and tail-suspension mice. **I.** Three-dimensional micro-CT reconstruction images of femurs from the control and tail-suspension mice. The *top panel* shows trabecular bone, and the *bottom panel* represents cortical bone. Representative examples are shown. **J.** Quantitative microarchitectural parameters of micro-CT: BV/TV, Tb.Th., Tb.N., Tb.Sp. and Ct.Th. Five pairs of control and tail suspension mice were included in the measurement. **K.** Representative images of dual calcein-alizarin red S labeling of trabecular bone from the control and tail-suspension mice acquired 1 week after tail-suspension treatment and quantification of the mineral apposition rate. **L.** Representative images of the immunofluorescence for p-STAT3 in trabecular bone from the control and tail suspension mice. ***n* **= 5 mice per group

We established a running-wheel model to study bone changes and metabolism under exercise (Fig. 1A). H&E staining showed increased trabecular bone mass after exercise (Fig. 1B). Compared with the control group, the exercise group showed increased trabecular bone mass (Fig. 1C), as confirmed by increased BV/TV. and Tb.N. and decreased Tb.Sp.; Ct.Th. did not change significantly (Fig. 1D). Increased bone mass may be due to increased bone formation, decreased bone resorption, or both. Calcein-alizarin red double-labeling revealed that mineral apposition rate (MAR) in the exercise group was heightened with respect to the control group (Fig. 1E). The results suggested that the exercise mice exhibited increased bone formation. Tartrate-resistant acid phosphatase (TRAP) staining displayed more osteoclasts in the exercise group than the control group (Fig. S1A). TRAP staining suggested that the exercise mice exhibited increased bone resorption. These results indicated that mechanical force induced an increase in bone mass* in vivo* through increased bone formation.

Previous studies demonstrated that STAT3 plays an important role in bone metabolism 14, 16. We then wondered whether STAT3 might play the same role under exercise. The increased number of p-STAT3^+^OPN^+^ cells in the exercise mice (Fig. 1F) implied that increased bone mass mediated by exercise might be closely related to STAT3 activity.

To further study the relationship between exercise and STAT3 activity, we conducted a tail suspension (TS) model to mimic exercise loss (Fig. 1G). H&E staining showed decreased bone mass in the TS group after exercise loss (Fig. 1H). Meanwhile, a significant decline was observed in BV/TV, Tb.N., and Ct.Th (Fig. 1J). Further, calcein-alizarin red double-labeling showed that exercise loss decreased MAR in the TS mice, which suggested that exercise loss inhibited bone formation (Fig. 1K). TRAP staining showed that exercise loss increased osteoclast number in the TS mice, suggesting that exercise loss increased bone resorption (Fig. S1B). Furthermore, a decrease in p-STAT3^+^OPN^+^ cells was observed in the femoral bones of exercise-loss mice (Fig. 1L).

**Figure 2:**
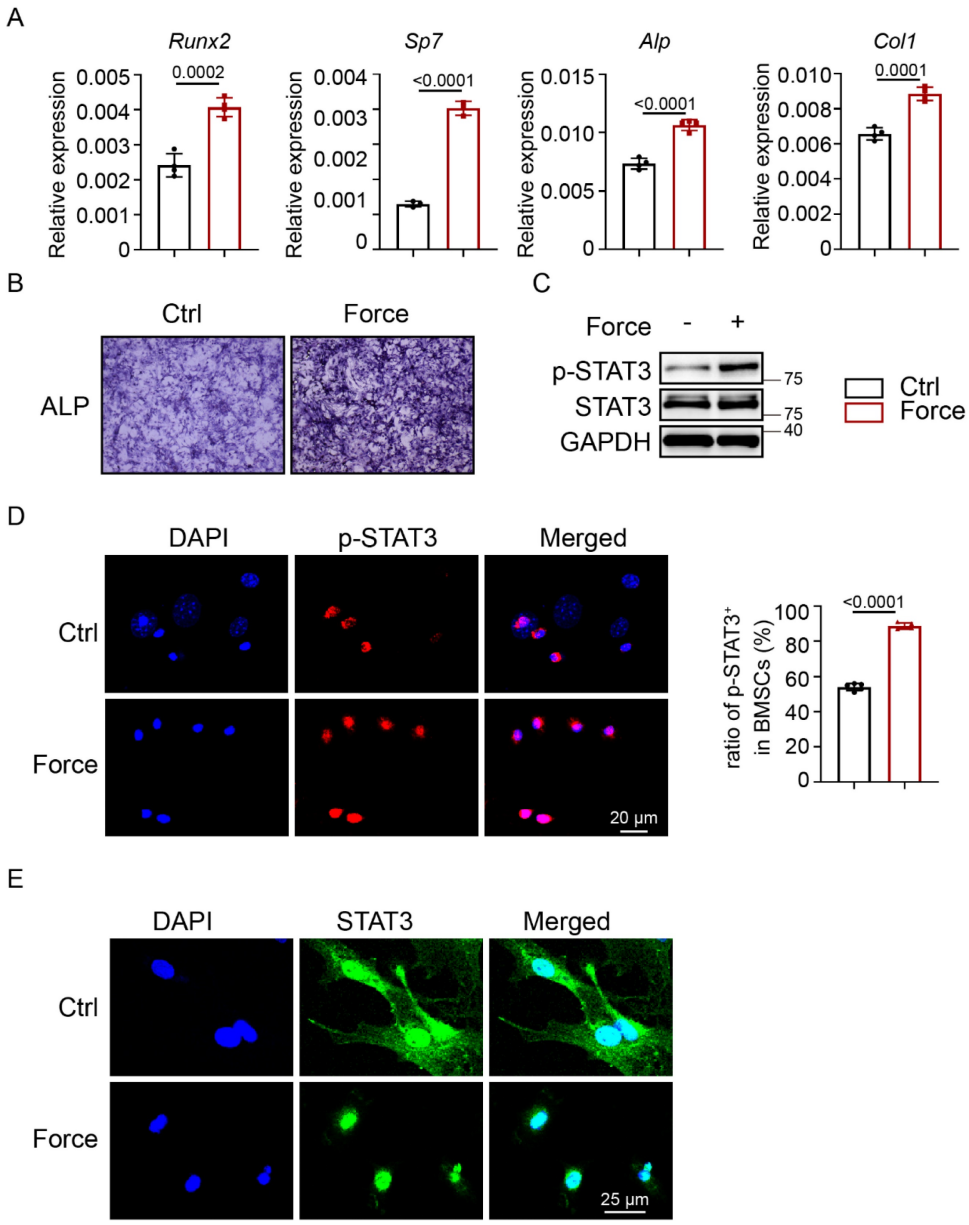
** Osteoblastic STAT3 was activated by mechanical force* in vitro*. A.** ALP staining of BMSCs after culture in osteogenic medium with or without mechanical force for 7 days. **B.** The relative mRNA levels of Runx2, Sp7, Alp, and Col1α1 in BMSCs with or without mechanical force for 7 days were quantified by qPCR. **C.** The expression of p-STAT3 and STAT3 in BMSCs with or without mechanical force for 8 h were analyzed by western blotting. **D.** Immunofluorescence staining of p-STAT3 in control and mechanical force-treated BMSCs for 8 h. **E.** Immunofluorescence staining of STAT3 in control and mechanical force-treated BMSCs for 8 h. Ratio of p-STAT3^+^ cells in control and mechanical force-treated BMSCs for 8 h.

To further investigate whether mechanical force could activate STAT3, the Flexcell tension system was applied to simulate cyclic mechanical strain (CMS) *in vitro*. After CMS, BMSCs showed increased ALP activity and higher expression levels of osteogenic-specific markers (Fig. 2A, B). According to western blotting, the expression of p-STAT3 was highly increased in BMSCs (Fig. 2C). This was in agreement with the significantly increased number of p-STAT3-positive BMSCs (Fig. 2D). STAT3 positive expression in BMSCs transferred from the cytoplasm to the nucleus after CMS (Fig. 2E). These results indicated that osteoblastic STAT3 activity might be closely related to exercise-induced osteogenesis and bone mass.

### Pharmacological inhibition of STAT3 impaired exercise-induced osteogenesis

**Figure 3:**
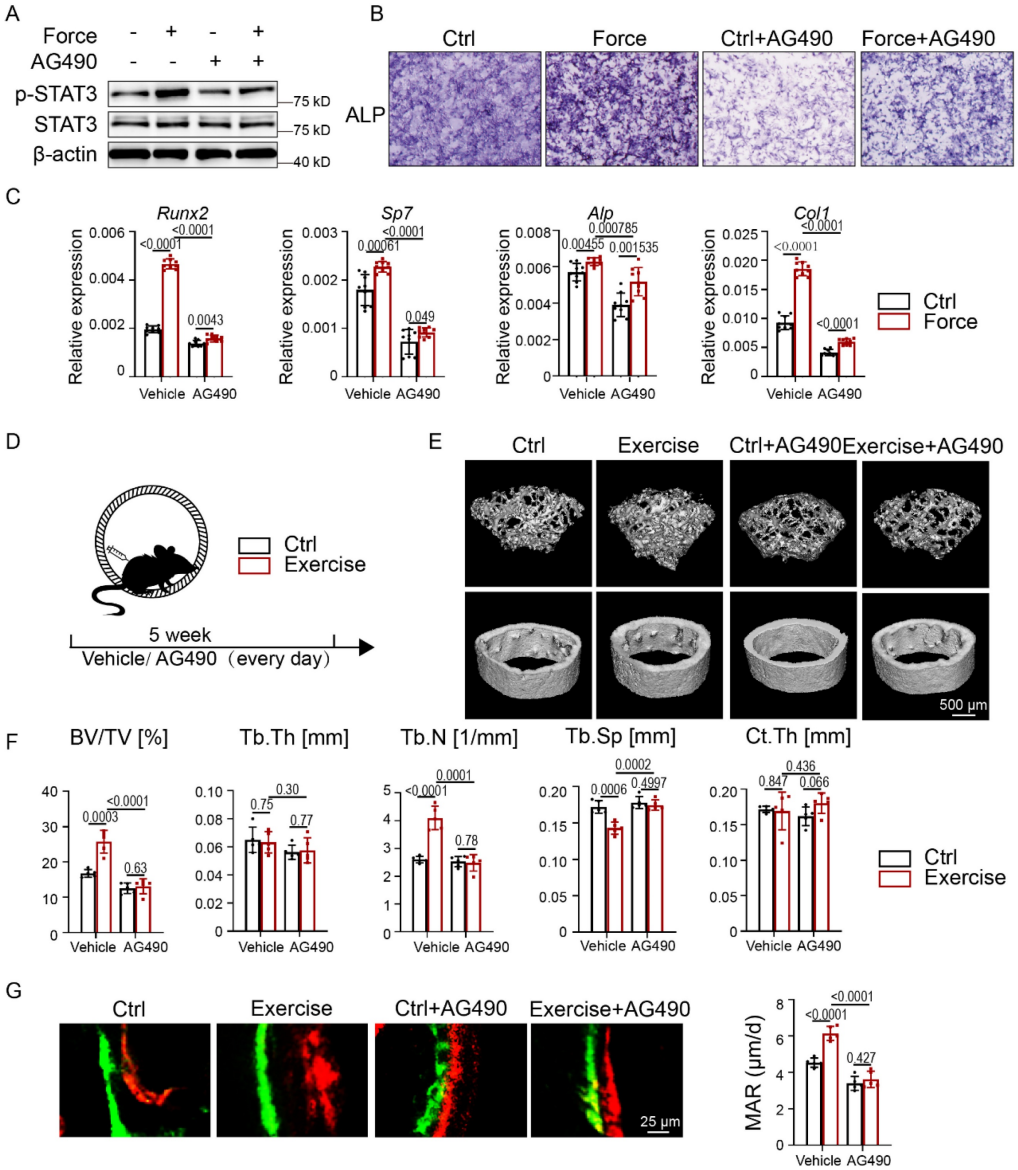
** Pharmacological inhibition of STAT3 impaired osteogenesis under mechanical force *in vitro* and *in vivo*. A.** Expressions of p-STAT3 and STAT3 in BMSCs with or without mechanical force/AG490 for 8 h were analyzed by western blotting. **B.** ALP staining of BMSCs after culture in osteogenic medium with or without mechanical force/AG490 for 7 days. **C.** The relative mRNA levels of *Runx2*, *Sp7*, *Alp*, and *Col1α1* in BMSCs with or without mechanical force/AG490 for 7 days were quantified by qPCR. **D.** Experimental outline of the running-wheel test with AG490 treatment. **E.** Three-dimensional micro-CT reconstruction images of femurs from the control and exercise mice treated with or without AG490. The *top panel* shows trabecular bone, and the *bottom panel* represents cortical bone. Representative examples are shown. **F.** Quantitative microarchitectural parameters of micro-CT: BV/TV, Tb.Th., Tb.N., Tb.Sp. and Ct.Th. Five pairs of control and exercise mice treated with or without AG490 were included in the measurement. **G.** Representative images of dual calcein-alizarin red S labeling of trabecular bone from the control and exercise mice treated with or without AG490 acquired 5 weeks after *in vivo* exercise treatment, and the quantification of the mineral apposition rate. ***n* **= 5 mice per group. (Paired Student's t test or 2-way ANOVA)

Since STAT3 is considered a promising anticancer target, we wondered whether pharmacological inhibition of STAT3 could regulate exercise mediated osteogenesis. Firstly, we cultured BMSCs under CMS with and without a classic JAK2-STAT3 inhibitor, AG490. Western blotting indicated that the phosphorylation of STAT3 was significantly inhibited in BMSCs treated with AG490 both with and without CMS (Fig. 3A). After treatment with AG490, BMSCs showed decreased ALP activity and reduced expression levels of osteogenic-specific markers both with and without CMS (Fig. 3B-C).

To further elucidate whether STAT3 is a suitable target for exercise-mediated osteogenesis *in vivo*, we conducted intraperitoneal injections of AG490 in the exercise model. With micro-CT analysis, we found that AG490 treatment in exercise mice induced bone loss as marked by decreased BV/TV and Tb.N. and increased Tb.Sp. compared with the exercise group. In addition, Ct.Th. did not change significantly (Fig. 3D-F). H&E staining showed decreased bone mass after AG490 treatment in both the control and exercise groups (Fig. S2A). Calcein-alizarin red double-labeling showed impaired MAR in the exercise group treated with AG490, suggesting that bone formation activity was attenuated with AG490 treatment in mice with exercise (Fig. 3G). TRAP staining showed less osteoclast number in AG490-treated exercise mice, exhibiting reduced bone resorption (Fig. S2B). Pharmacological inhibition of STAT3 could decelerate skeletal development and interrupt force-mediated bone homeostasis, suggesting that STAT3 might be a suitable target for mechanical force-mediated disease.

### Osteoblast lineage-specific deletion of STAT3 impaired exercise-induced osteogenesis

**Figure 4:**
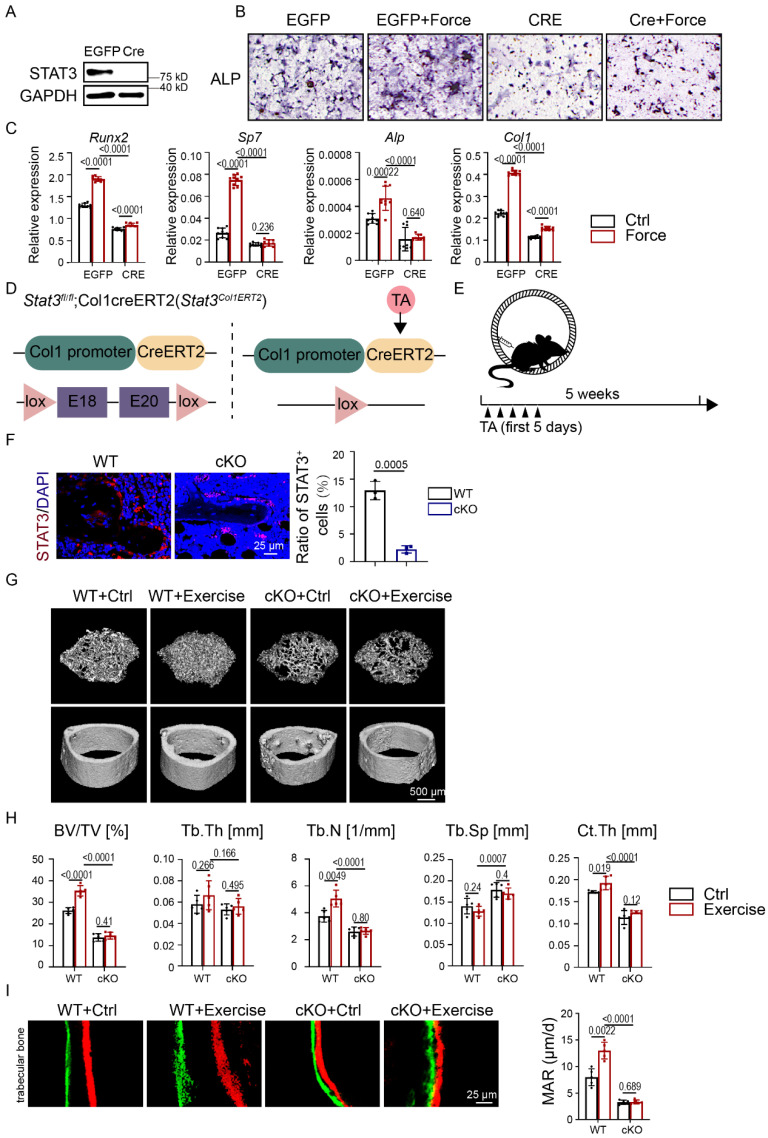
** Osteoblast lineage-specific deletion of STAT3 impaired osteogenesis under mechanical force *in vitro* and *in vivo*. A.** Expression of STAT3 in BMSCs infected with adenovirus expressing EGFP or Cre-EGFP for 48 h was analyzed by western blotting. **B.** ALP staining of BMSCs after culture in osteogenic medium with or without mechanical force/*Stat3* knockout for 7 days. **C.** The relative mRNA levels of *Runx2*, *Sp7*, *Alp*, and *Col1α1* in BMSCs with or without mechanical force/*Stat3* knockout for 7 days were quantified by qPCR. **D.** Illustration of inducible *Stat3* deletion in Col1a1-expressing osteoblast lineage cells. **E.** Experimental outline of the running-wheel test with inducible *Stat3* deletion in Col1a1 lineage cells.** F.** Immunofluorescence staining of STAT3 in the control and *Stat3*-knockout mice. **G.** Three-dimensional micro-CT reconstruction images of femurs from the control and exercise mice treated with or without *Stat3* knockout. The *top panel* shows trabecular bone, and the *bottom panel* represents cortical bone. Representative examples are shown.** H.** Quantitative microarchitectural parameters of micro-CT: BV/TV, Tb.Th., Tb.N., Tb.Sp. and Ct.Th. Five pairs of control and exercise mice treated with or without *Stat3* knockout were included in the measurement. **I.** Representative images of dual calcein-alizarin red S labeling of trabecular bone from the control and exercise mice treated with or without *Stat3* knockout acquired 5 weeks after *in vivo* exercise treatment, and the quantification of the mineral apposition rate. ***n* **= 5 mice per group. (Paired Student's t test or 2-way ANOVA)

To further explore the effect of STAT3 on osteoblast differentiation of mechanical force-exposed BMSCs, we infected BMSCs from *Stat3^fl/fl^* mice with adenovirus expressing GFP (Ad-EGFP) and CRE recombinase (Ad-CRE) to knock out *Stat3*. Western blotting implied the ablation of STAT3 in BMSCs (Fig. 4A). After CMS, the Ad-CRE group showed decreased ALP activity compared with the Ad-EGFP group (Fig. 4B). The mRNA expression of the osteogenic markers was consistent with ALP staining (Fig. 4C).

We next applied tamoxifen-inducible osteoblast lineage cells expressing cre, Col1ERT2 cre to induce osteoblast lineage-specific *Stat3* knockout during exercise (Fig. 4D). Immunofluorescence analysis confirmed the deletion of STAT3 in the osteoblast lineage in *Stat3*^Col1ERT2^ mice (Fig. 4F). Consistent with mice treated with AG490, the micro-CT and H&E staining results showed that the trabecular bone mass was significantly reduced in the *Stat3*^Col1ERT2^ mice compared to *Stat3*^fl/fl^ both with and without exercise, as confirmed by decreased BV/TV and Tb.N.; Tb.Th., Tb.Sp., and Ct.Th. did not change significantly (Fig. 4G-H, Fig. S3A). Moreover, there was no difference in the *Stat3*^Col1ERT2^ mice in both the control and exercise groups, underlying the vital role of osteoblast STAT3 under mechanical force. Double-labeling showed decreased MAR in the *Stat3*^Col1ERT2^ mice, suggesting that bone formation activity was abrogated in the absence of STAT3 (Fig. 4I). TRAP staining exhibited fewer osteoclasts in the *Stat3*^Col1ERT2^ mice, implying that bone resorption activity declined in the absence of STAT3 (Fig. S3B). All these results indicated that mechanosensitive STAT3 plays a crucial role in exercise-mediated osteogenesis.

### Pharmacological activation of STAT3 ameliorated bone loss due to exercise loss by promoting osteogenesis

**Figure 5:**
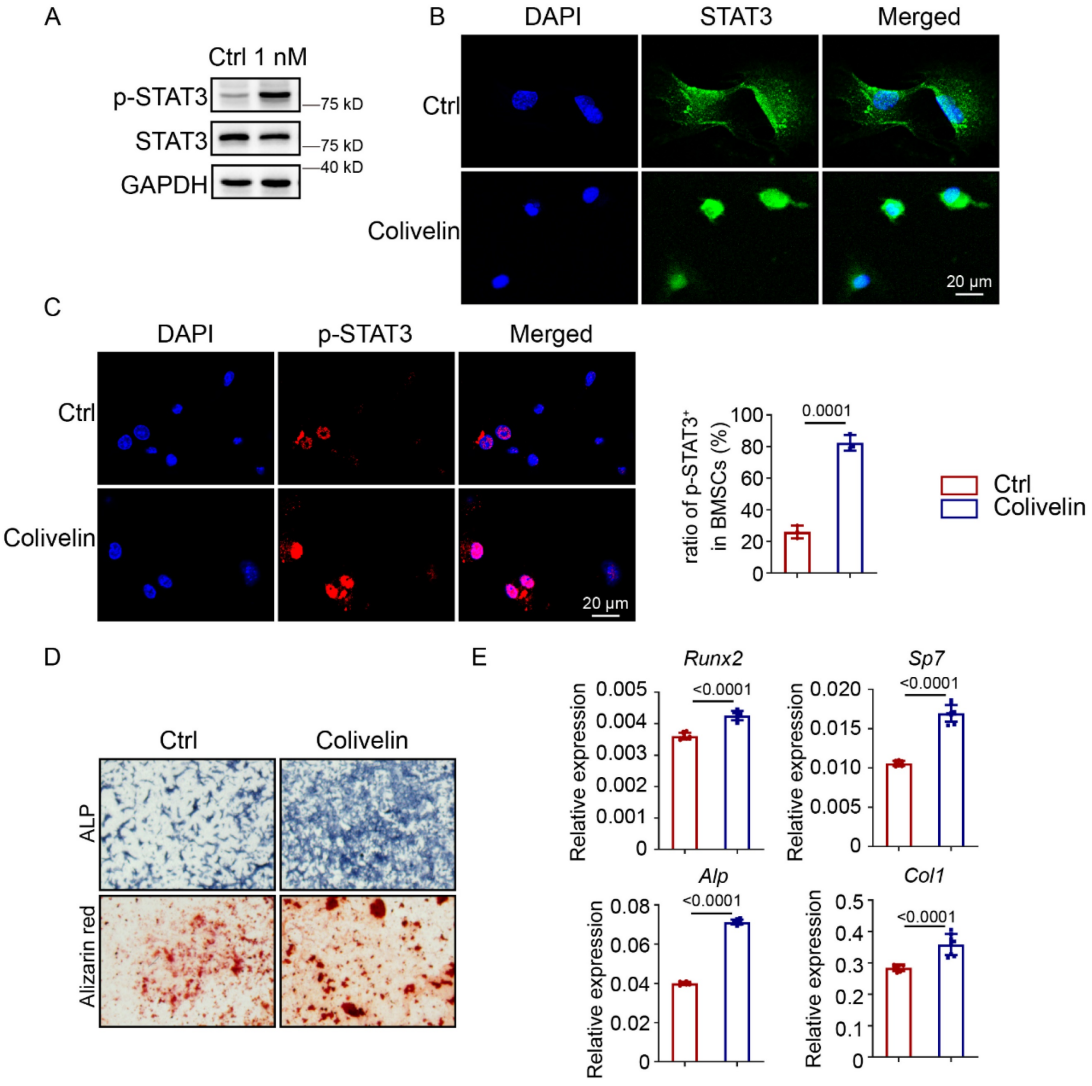
** Pharmacological activation of STAT3 activity promoted osteoblast differentiation. A.** Expression of p-STAT3 and STAT3 in BMSCs with or without colivelin for 12 h were analyzed by western blotting. **B.** Immunofluorescence staining of STAT3 in control and colivelin-treated BMSCs treated for 12 h. **C.** Immunofluorescence staining of p-STAT3 in control and colivelin-treated BMSCs treated for 12 h. The ratio of p-STAT3^+^ cells in the control and colivelin treated groups. **D.** ALP staining and alizarin red S staining of BMSCs after culture in osteogenic medium with or without colivelin for 7 and 14 days separately. **E.** The relative mRNA levels of *Runx2*, *Sp7*, *Alp,* and *Col1α1* in BMSCs with or without colivelin for 7 days were quantified by qPCR.

Since mechanical force could encourage STAT3 phosphorylation, we supposed that activation of STAT3 could promote osteoblast differentiation and maintain bone homeostasis in the absence of mechanical force. To test this hypothesis, we first analyzed the protein level of p-STAT3 in BMSCs treated with colivelin, a STAT3 agonist. Western blotting indicated that colivelin treatment resulted in higher levels of p-STAT3 compared with the control group (Fig. 5A). After colivelin treatment, positive expression of STAT3 in BMSCs transferred from the cytoplasm to the nucleus (Fig. 5B), and the number of p-STAT3-positive BMSCs increased (Fig. 5C). Then, we discovered that colivelin encouraged osteogenic differentiation, as indicated by increased ALP activity and calcified nodule formation (Fig. 5D). The expression of osteogenic markers was also boosted after treatment (Fig. 5E).

**Figure 6:**
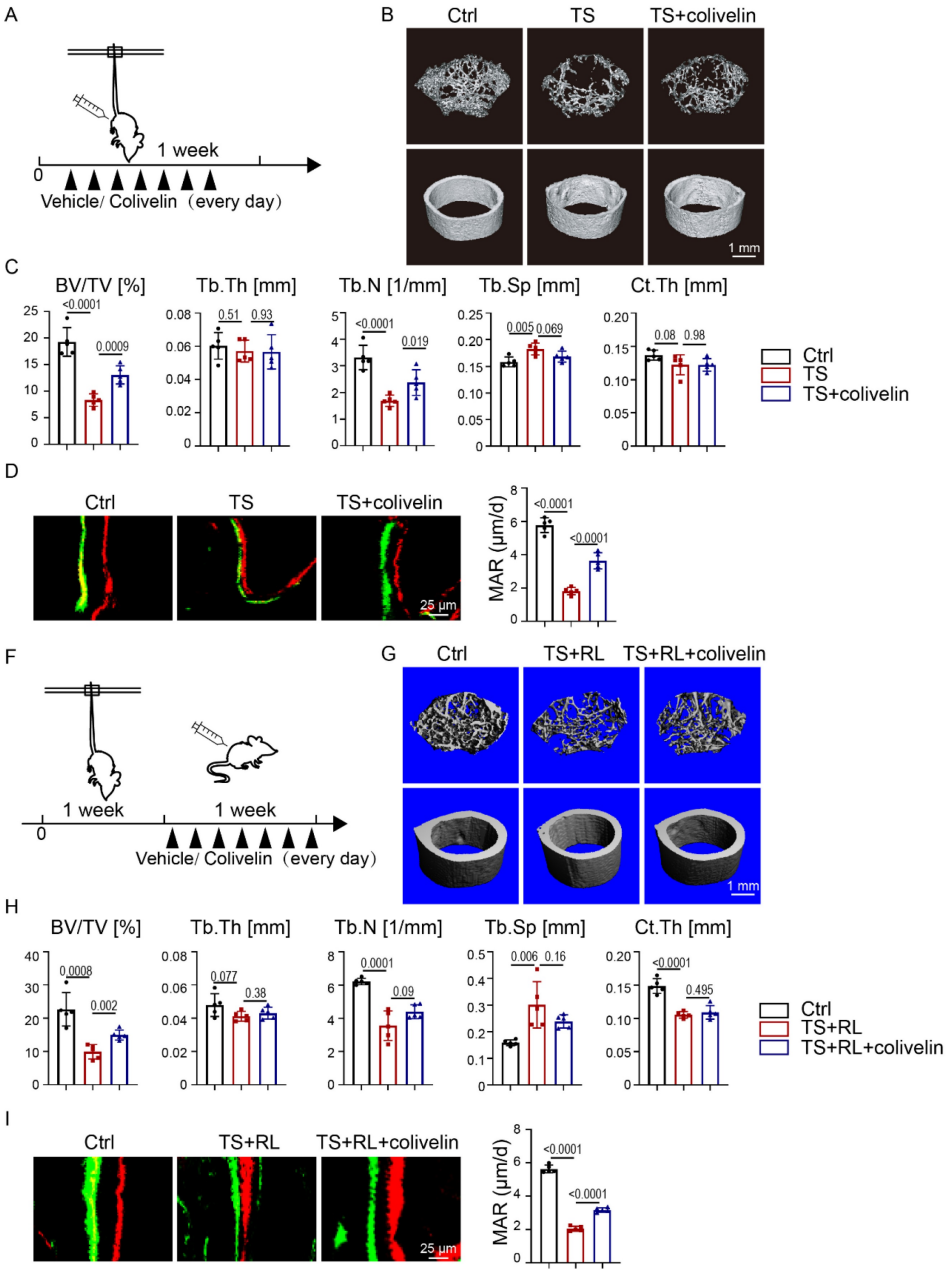
** Pharmacological activation of STAT3 promoted osteogenesis in the absence of mechanical force *in vivo*. A.** Experimental outline. **B.** Three-dimensional micro-CT reconstruction images of femurs from the control and tail-suspension mice with or without colivelin treatment. The *top panel* shows trabecular bone, and the *bottom panel* represents cortical bone. Representative examples are shown. **C.** Quantitative microarchitectural parameters of micro-CT: BV/TV, Tb.Th., Tb.N., Tb.Sp. and Ct.Th. Five pairs of control and exercise mice treated with or without *Stat3* knockout were included in the measurement. **D.** Representative images of dual calcein-alizarin red S labeling of trabecular bone from the control and exercise mice treated with or without *Stat3* knockout acquired 5 weeks after *in vivo* exercise treatment and the quantification of the mineral apposition rate. **E.** Representative images of TRAP staining of trabecular bone from the control and exercise mice with or without *Stat3* knockout. **F.** Experimental outline. **G.** Three-dimensional micro-CT reconstruction images of femurs from the control and tail-suspension mice with or without colivelin treatment. The *top panel* shows trabecular bone, and the *bottom panel* represents cortical bone. Representative examples are shown. **H.** Quantitative microarchitectural parameters of micro-CT: BV/TV, Tb.Th., Tb.N., Tb.Sp. and Ct.Th. Five pairs of control and exercise mice treated with or without *Stat3* knockout were included in the measurement. **I.** Representative images of dual calcein-alizarin red S labeling of trabecular bone from the control and exercise mice treated with or without *Stat3* knockout acquired 5 weeks after *in vivo* exercise treatment, and the quantification of the mineral apposition rate. ***n* **= 5 mice per group.

To further investigate whether colivelin could maintain bone homeostasis in the absence of exercise, a TS model was performed (Fig. 6A). As indicated by micro-CT analysis (Fig. 6B, C), the BV/TV and Tb.N. of trabecular bone in colivelin-treated TS mice were statistically significantly increased compared with the TS mice, implying that colivelin treatment could relatively rescue bone loss. Calcein-alizarin red double-labeling showed increased MAR in colivelin-treated TS mice compared with TS mice, suggesting that colivelin promoted bone formation under exercise loss (Fig. 6D). TRAP staining showed no difference between the TS group treated or not with colivelin (Fig. S4A). These results fully illustrated that pharmacological activation of STAT3 could be used to prevent osteoporosis caused by the loss of exercise.

We next sought to examine the therapeutic effect of colivelin after disuse bone loss (Fig. 6F). Mice got reloaded after 1-week-tail-suspension. As shown by micro-CT analysis, colivelin increased the BV/TV and Tb.N. of trabecular bone after bone loss, indicating that the recovery rate of bone loss was accelerated by the activation of STAT3 (Fig. 6G, H). Calcein-alizarin red double-labeling suggested that colivelin promoted bone formation (Fig. 6I). TRAP staining showed there was no significant difference in osteoclast number between the TS group treated with and without colivelin (Fig. S4B). All these data suggested that STAT3 might act as a suitable pharmacological target for the treatment of disuse bone loss.

## Discussion

Our current study demonstrated that STAT3 serves as a mechanotransduction regulator in bone homeostasis both *in vivo* and *in vitro*. Pharmacological inhibition and osteoblast lineage-specific ablation of STAT3 interrupted force-mediated bone homeostasis by decreasing bone formation. Meanwhile, pharmacological activation of STAT3 promoted osteoblast differentiation and rescued bone loss in the absence of exercise. Thus, STAT3 may act as a suitable pharmacological target for the treatment of bone metabolic diseases in the future.

As stated by Wolff's law in 1892, bone changes under exercise 1. Without exercise loading, a newborn showed osteopenia and mechanical defects 19. Also, astronauts have shown bone loss 20. Exercise-mediated mechanical force is vital for maintaining bone homeostasis. However, the underlying mechanism remains unknown. Recent studies demonstrated that mechanical signals regulate the balance between bone, muscle, and fat 21. However, the mechanical signals from physical to physiological are unclear. Here, a running-wheel exercise model and a Flexcell tension system were used to mimic mechanical loading *in vivo* and *in vitro* 22. BMSCs are mechanosensitive cells that coordinate bone resorption and formation 7. In the present study, exercise promoted the accumulation of bone mass and metabolism by osteogenesis and CMS promoted osteoblastic differentiation in BMSCs. On the contrary, the tail suspension model was used to imitate exercise loss *in vivo.* Our experiments suggested that exercise loss reduced bone mass by decreasing bone formation and increasing bone resorption.

STAT3, as one of the STAT family members, takes part in many critical physiological processes 23-25. Our previous study demonstrated that STAT3 plays an essential role in bone homeostasis, which controls both osteoblast and osteoclast differentiation 14, 16. Recent studies showed that STAT3 was phosphorylated in response to mechanical loading in periodontal ligament cells and osteocytes and so on 26-30, and *Stat3* in osteocytes was indicated to mediate osteogenic response to loading 29. Others also demonstrated that load-induced bone formation may be increased by augmenting STAT3 signaling within osteocytes 31. The osteoblastic STAT3 in BMSCs under mechanical force remains unclear. In the present study, the immunofluorescence staining and western blotting analysis confirmed that STAT3 is mechanosensitive in BMSCs. We further inhibited STAT3 from phosphorylation and knocked out *Stat3* in BMSCs *in vivo* and *in vitro*, which impaired osteogenesis and bone homeostasis, which indicated the STAT3 plays a significant part in osteogenesis. Furthermore, there was no significant difference between the control group and force group with inhibition or knocked out of STAT3, underlying the key role of STAT3 in osteogenesis mediated by exercise. Our data showed that exercise-mediated mechanical force could activate STAT3 signals in osteoblast lineage cells.

Meanwhile, STAT3 is considered an imperative anticancer target 32, 33. STAT3 inhibitors have long been widely used in the treatment of cancers 34-37. Recently, STAT3 has been more appreciated in bone-related diseases 38, 39. AG490, a tyrosine kinase inhibitor that inhibits the activation of Stat-3 by selectively blocking JAK2, is widely used in antitumor therapy research 26, 40, 41. In our study, AG490 was verified to inhibit STAT3 activity through western blot. *In vivo* data demonstrated the inhibition of osteoblastic STAT3 could prevent osteogenesis, confirming the key role of STAT3 in bone homeostasis. Further inhibition of osteoblastic STAT3 under exercise-mediated mechanical force underlined that force promoted osteogenesis *via* mechanosensitive STAT3. Also, colivelin, a potent STAT3 agonist, was used to activate STAT3 under exercise loss. The activation of STAT3 partly rescued bone loss under exercise loss, which could be used to prevent and treat disuse bone loss. Our study verified that STAT3 is a suitable therapeutic target for force-induced bone disease.

In conclusion, our study has refined a critical role of STAT3 in exercise-mediated osteogenesis and revealed a potential therapeutic strategy for exercise loss osteoporosis.

## Materials and Methods

### Ethics statement

This study complied with all relevant ethical regulations for animal testing and research. All experimental animal procedures were approved by the Institutional Animal Care and Research Advisory Committee of the Shanghai Ninth People's Hospital, School of Medicine, Shanghai Jiao Tong University and were performed according to the institutional guidelines (approval number: HKDL[2018]386) and the ARRIVE guidelines (Animal Research: Reporting *In vivo* Experiments). All mice were bred and maintained under specific pathogen free (SPF) conditions.

### Mice

*Stat3*^fl/fl^ mice were purchased from the Jackson Laboratory (No. 016923). *Stat3*^fl/fl^ were crossed with Col1ERT2-cre mice (Col1ERT2-cre; provided by Bin Zhou, Shanghai Institute for Biological Sciences, Chinese Academy of Sciences) to generate *Stat3*^fl/fl^; Col1-creERT2 mice (hereafter called *Stat3*^Col1ERT2^). All mice in this study were maintained on the C57BL/6 background. All these mice were bred and maintained under specific pathogen-free (SPF) conditions.

#### Exercise model

Twelve 4-week-old male WT C57ML/6 mice were randomly divided into two groups: the control group and exercise group. The mice in the exercise group were individually housed in cages and had free access to a stainless-steel running wheel (14 cm outer diameter) daily for 5 weeks. The mice in the control group were housed individually without a running wheel. Bilateral femurs and tibiae were dissected for analysis.

#### Tail suspension model

Twelve 4-week-old male WT C57ML/6 mice were randomly divided into two groups: the control group and tail suspension (TS) group. The mice in the TS group were suspended in individual cages for 24 hours a day for 7 days at an approximate 45-degree head-down tilt. Their hind limbs were able to touch the ground, and the mice could get food.

#### Histological staining

Femurs were fixed in 4% paraformaldehyde for 24 h and decalcified in 15% EDTA for 1 month. The specimens were embedded in paraffin and sectioned at 8 μm. Tissue sections were used for H&E staining, tartrate-resistant acid phosphatase (TRAP) staining, immunohistochemical staining, and immunofluorescence staining.

H&E staining (Beyotime, C0105M) and TRAP staining (Sigma, 387A-1KT) were performed after sections were dewaxed and dehydrated. Images were captured with a microscope.

#### Micro-quantitative computed tomography analysis

Femurs were fixed in 4% paraformaldehyde for 24 h and then scanned with a Quantum GX2 (PerkinElmer, Waltham, USA) instrument. The turn-on voltage was 80 kV, and the current density was 88 μA. A total 900 μm width of trabecular bone below the distal growth plate was three-dimensionally reconstructed and analyzed for microarchitectural parameters of BV/TV, Tb.Th., Tb.N., and Tb.Sp. Meanwhile, a 900-μm wide section of cortical bone from the middle of the femur was analyzed for Ct.Th.

#### Calcein-alizarin red S labeling

Mice were intraperitoneally injected with calcein (Sigma, C0875-5G, 20 mg/kg) and alizarin red S (Sigma, A5533-25G, 40 mg/kg) 5 days and 2 days before they were sacrificed. The femurs were harvested, fixed, dehydrated, embedded in polymethylmethacrylate, and cut into 10-μm sections. Fluorescence-labeled images were captured with a microscope. The MAR was calculated as the average distance between two labels divided by the time interval between two injections.

#### Immunofluorescence

Paraffin sections were dewaxed in xylene and rehydrated in graded alcohol solutions. Then, sections were blocked in PBS with 10% horse serum for 1 h and then stained overnight at 4 °C with antibodies against p-STAT3 (CST, #9138, mouse monoclonal, 1:200), OPN (R&D, AF808, 1:1000), and STAT3 (Santa Cruz, sc-482, rabbit polyclonal, 1:50). The next day, the sections were washed and incubated with relevant secondary antibody at room temperature. DAPI (Sigma, D8417) was used for counterstaining.

For cellular immunofluorescence, cells were seeded on a coverglass and then fixed with 4% paraformaldehyde for 10 min. Subsequent steps were the same as those used for tissue immunofluorescence. Antibodies against p-STAT3 (CST, #9138, mouse monoclonal, 1:200) and STAT3 (Santa Cruz, sc-482, rabbit polyclonal, 1:50) and the relevant secondary antibodies were used. Images were captured with a microscope.

#### Cell culture

BMSCs were isolated from the femurs and tibias of 4-week-old C57BL/6 mice. The bone marrow was flushed with a 5-ml syringe of Minimum Essential Medium-α (α-MEM, Corning, 10-022-CVR) and then cultured in α-MEM with 10% fetal bovine serum (Ausbian, WS500T) and 1% penicillin-streptomycin (Thermo Fisher Scientific, No. 15140122) at 37 °C in 5% CO2. The medium was renewed every 3 days until BMSCs reached nearly 80% confluence.

For osteoblast differentiation, BMSCs were seeded in different plates and cultured in an osteogenic medium (Cyagen, MUBMD-90021). ALP staining (Beyotime, P0321S) was performed after a 7-day induction. Alizarin red S staining (Cyagen, MUBMD-90021) was performed after a 14-day induction as previously described 14.

For AG490 (MCE, HY-12000) treatment, BMSCs were seeded in BioFlex plates at a density of 2.0 × 10^5^. The changed medium was treated with 20mM AG490, and after 8 h the protein was extracted for western blotting.

For adenovirus (Hanbio Biotechnology Co. Ltd) treatment, BMSCs were isolated from *Stat3*^fl/fl^ mice and seeded in BioFlex plates at a density of 2.0 × 10^5^. After 24 h, the cells were infected with adenovirus expressing either CRE recombinase or GFP at a multiplicity of infection of 50.

For colivelin (MCE, HY-P1061A) treatment, BMSCs were seeded in 96-well plates at a density of 1.0 × 105. The changed medium was treated with 1 nM colivelin and after 1 h, the protein was extracted for western blotting.

#### Mechanical compression device

BMSCs were seeded into collagen I-coated BioFlex plates at a density of 2.0 × 105. The mechanical compression system (Flexcell-4000) was set according to the instructions. A definite condition of 10% elongation, 0.5 Hz, and 8 h was applied to the cells as previously described 42.

#### RT-PCR analysis

RNA was obtained from osteoblasts using TRIzol (Sigma, T9424) and was reverse-transcribed into cDNA using the PrimeScript RT master kit (TakaRa Bio Inc., RR036A). Real-time reverse transcription PCR was performed using the Bio-Rad CFX96 system. The primer sets used were as follows:

#### Western blotting

Proteins were obtained from osteoblasts using lysis buffer (TaKaRa, No. 9173) containing protease and phosphatase inhibitors. The follow-up operation abided with standard protocols. Anti-STAT3 (Santa Cruz, sc-482, rabbit polyclonal, 1:200), anti-p-STAT3 (CST, #9138, mouse monoclonal, 1:1000), anti-GAPDH (CST, #2118, rabbit monoclonal, 1:100000), and anti-β-actin (CST, #4970, rabbit monoclonal, 1:100000) were used.

#### Mice treated with AG490

Mice were randomly allocated into two groups. Half the mice received daily intraperitoneal injections of PBS for 5 weeks. Half the mice received daily intraperitoneal injections of 5 mg/kg AG490 for 5 weeks.

#### Mice treated with colivelin

Mice were randomly allocated into two groups. Half the mice received daily intraperitoneal injections of PBS. Half the mice received daily intraperitoneal injections of 1 mg/kg colivelin for 1 week.

### Statistical analysis

Data are expressed as the mean ± standard deviation (SD). All experiments were repeated at least 3 times, and representative experiments are shown. Student's t tests were performed to evaluate the differences between the experimental and control groups. Comparisons of multiple groups were made using a 1- or 2-way ANOVA. p < 0.05 was considered statistically significant. Statistical analysis was conducted using SPSS and GraphPad Prism.

## Supplementary Material

Supplementary figures.

## Figures and Tables

**Table 1:** Primers

Primers for RT qPCR	
Hprt-RT-F	GTTAAGCAGTACAGCCCCAAA
Hprt-RT-R	AGGGCATATCCAACAACAAACTT
Runx2-RT-F	GGCCGGGAATGATGAGAACTAC
Runx2-RT-R	GGACCGTCCACTGTCACTTT
Sp7-RT-F	CCTTCCCTCACTCATTTCCTGG
Sp7-RT-R	TGTTGCCTGGACCTGGTGAGAT
ALP-RT-F	CGGGACTGGTACTCGGATAA
ALP-RT-R	ATTCCACGTCGGTTCTGTTC
Col1a1-RT-F	GCTCCTCTTAGGGGCCACT
Col1a1-RT-R	CCACGTCTCACCATTGGGG
