# Supplementary material for: Exercise maintains bone homeostasis by promoting osteogenesis through STAT3
Source: Int J Biol Sci. 2023 Apr 2;19(7):2021–33. doi: 10.7150/ijbs.82744 (PMC10158023; doi:10.7150/ijbs.82744)
Supplement: Supplementary file 1 — Supplementary figures. [file ijbsv19p2021s1.pdf]

Supplementary Figures

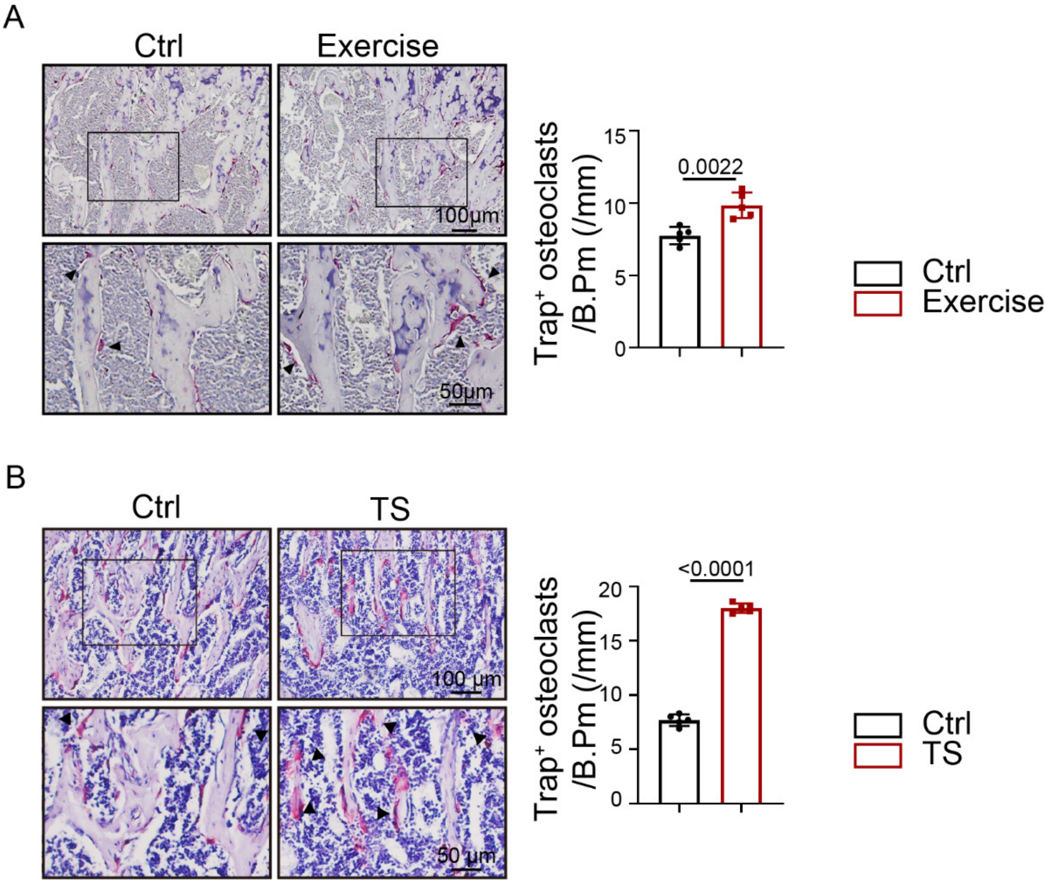

Figure S1.

A. Representative images of TRAP staining of trabecular bone from the control and exercise mice.

B. Representative images of TRAP staining of trabecular bone from the control and tail-suspension mice

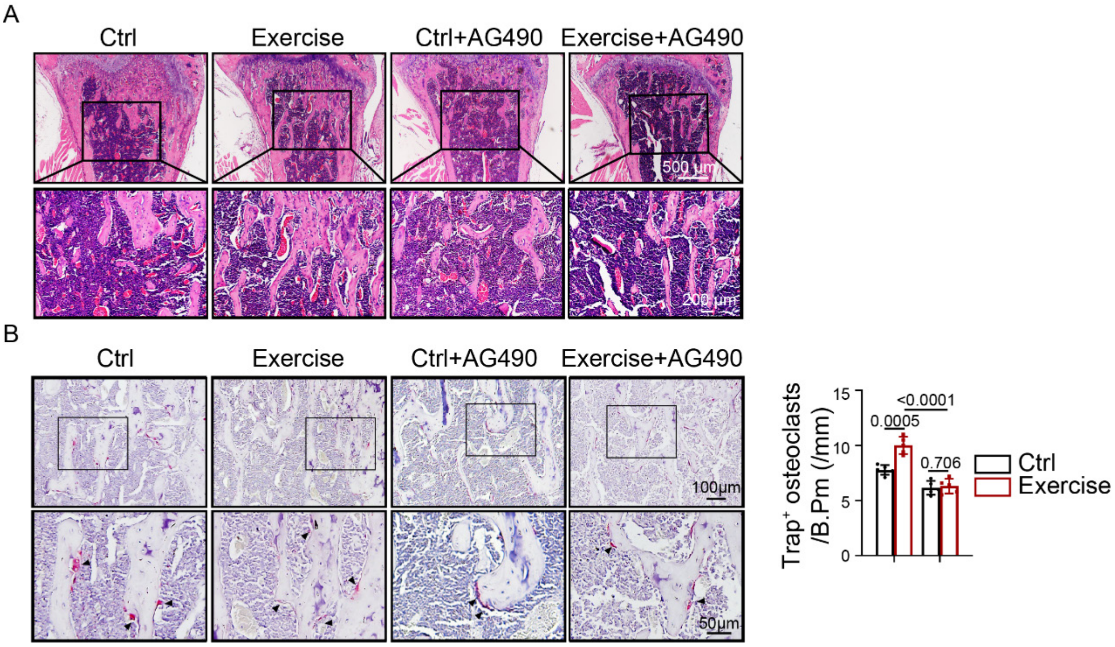

Figure S2.

A. H&E staining of the femurs of the control and exercise mice treated with or without AG490.

B. Representative images of TRAP staining of trabecular bone from the control and exercise mice with or without AG490.

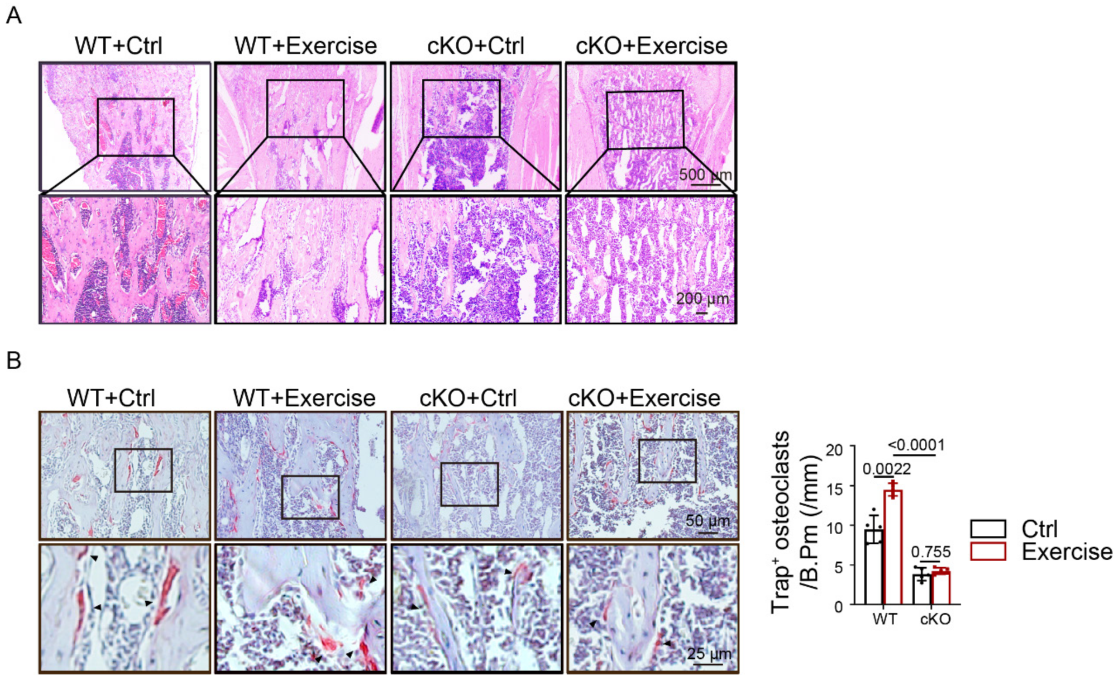

Figure S3.

A. H&E staining of the femurs of the control and exercise mice.

B. Representative images of TRAP staining of trabecular bone from the control and exercise mice with or without *Stat3* knockout.

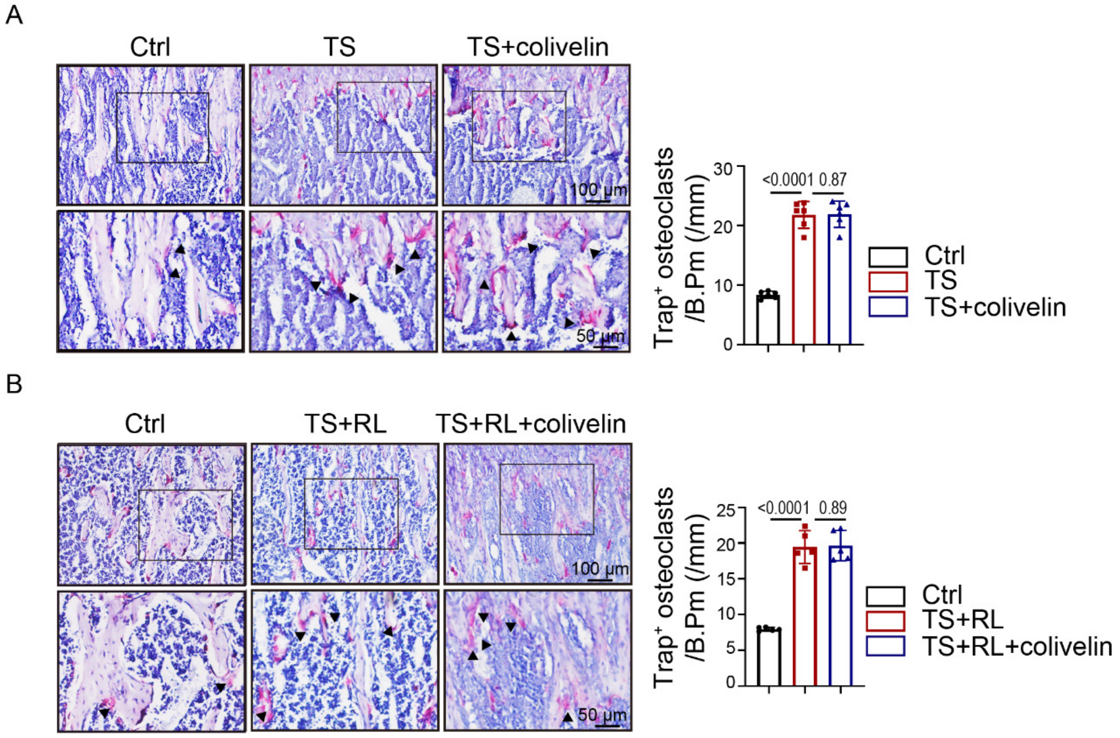

Figure S4.

A. Representative images of TRAP staining of trabecular bone from the control and tail-suspension mice with or without colivelin treatment.

B. Representative images of TRAP staining of trabecular bone from mice with or without colivelin treatment after tail suspension.
